# Spondylarthrite ankylosante et maladie de Still: un lien physiopathologique ou une simple association?

**DOI:** 10.11604/pamj.2017.28.132.5607

**Published:** 2017-10-10

**Authors:** Nassira Aradoini, Sofia Talbi, Hamida Azzouzi, Fatima Zahra Abourazzak, Hajar Khibri, Rhizlane Berrady, Wafaa Bono, Taoufik Harzy

**Affiliations:** 1 Faculté de Médecine, Université Sidi Mohamed Ben Abdallah, Service de Rhumatologie, Centre Hospitalier Universitaire Hassan II, Fès, Maroc; 2 Service de Médecine Interne, Centre Hospitalier Universitaire Hassan II, Fès, Maroc

**Keywords:** Spondylarthrite ankylosante, Still, fièvre, glucocorticoïdes, Ankylosing spondylitis, Still, fever, glucocorticoids

## Abstract

La spondylarthrite ankylosante est un rhumatisme inflammatoire chronique,qui fait partie des groupes de spondyloarthrites, au cours duquel les signes généraux comme la fièvre et l'amaigrissement sont peu importants. La maladie de Still de l'adulte est une affection systémique rare, qui reste un diagnostic d'élimination, et qui associe classiquement une fièvre élevée hectique, une éruption cutanée, des arthrites, et des manifestations systémiques diverses. Peu de cas de spondylarthrites ankylosantes présentés avec un tableau de la maladie de Still de l'adulte ont été décrits dans la littérature. Nous rapportons un nouveau cas d'un patient de 31ans suivi pour spondylarthrite ankylosante et qui se présente avec une fièvre au long cours et des signes clinico-biologiques entrant dans le cadre de la maladie de Still de l'adulte. Un éventuel lien physiopathologique entre les deux pathologies peut être évoqué, même si leur survenue simultanée est rarement rapportée dans la littérature.

## Abstract (en)

Ankylosing spondylitis is a chronic inflammatory rheumatism; it is part of the group of spondyloarthrites. General signs such as fever and weight loss are of little importance. Adult Still’s disease is a rare systemic condition, a diagnosis of exclusion commonly characterized by high hectic fever, rash, arthritis and various systemic manifestations. Few cases of ankylosing spondylitis associated with adult Still’s disease have been described in the literature. We here report the case of a 31-year old patient followed up for ankylosing spondylitis presenting with fever which had lasted for a long time and clinico-biological signs compatible with adult Still’s disease. A possible pathophysiologic link between the two diseases may be suggested, even if their simultaneous occurrence has been rarely reported in the literature.

## Introduction

La spondylarthrite ankylosante est un rhumatisme inflammatoire chronique au cours duquel les signes généraux comme la fièvre et l'amaigrissement sont peu importants [1], et peuvent entrer dans le cadre d'une autre affection associée. Dans le cas de notre observation, les signes généraux entrant dans le cadre de la maladie de Still de l'adulte. La maladie de Still de l'adulte est une affection systémique rare, reste un diagnostic d'élimination, et associe classiquement une fièvre élevée hectique, une éruption cutanée, des arthrites, et des manifestations systémiques diverses. L'association de spondylarthrite ankylosante et de maladie de Still est rare. Quelques observations ont été rapportées dans la littérature. Cette association nous pousse à penser à un lien physiopathologique entre les deux pathologies.

## Patient et observation

**Figure 1:**
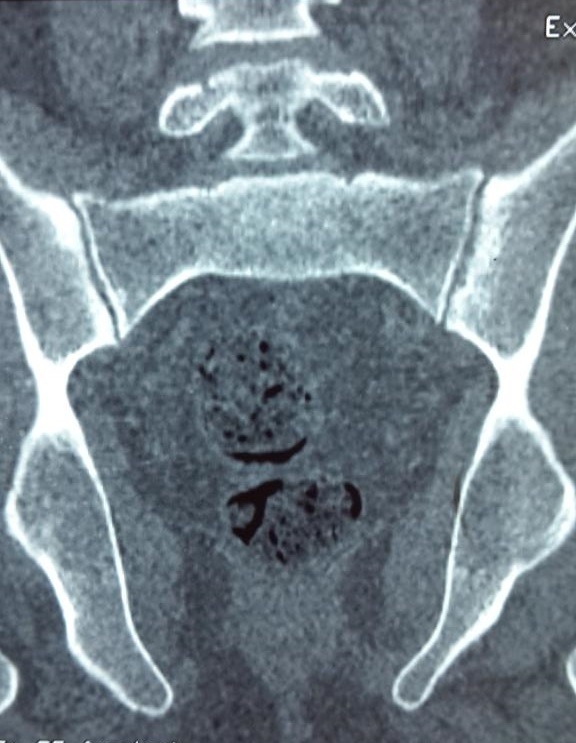
TDM du bassin, coupe frontale, objectivant une sacroiliite avec condensation des berges et érosions

**Figure 2:**
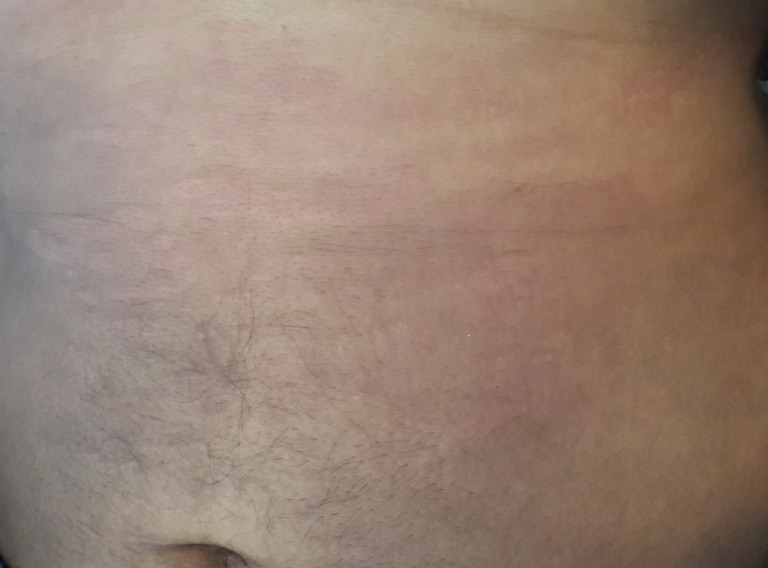
Rash cutané rose saumon maculo-papuleux et prurigineux

Un homme de 31 ans, suivi pour une spondyloarthrite à prédominance axiale évoluant depuis 10 ans, le diagnostic a étéretenu selon les critères ASAS [2], avec la présence d'une sacroiliite à la tomodensitométrie du bassin (Tableau 1), des rachialgies inflammatoires remontant à plus de 3 mois, des arthrites asymétriques prédominant au membres inférieurs, et une enthésite faite de talalgie inflammatoire. Il a été mis sous plusieurs classes d'anti-inflammatoires non stéroïdiens (ketoprofene, indométacine, celecoxib) à dose pleine avec efficacité partielle. Le patient soufrait depuis 1 mois d'une polyarthrite bilatérale asymétrique des grosses et des petites articulations (les mains, les poignets, et les genoux), associée à des lombalgies inflammatoires, évoluant dans un contexte de fièvre et d'altération de l'état général. L'examen clinique révélait en plus des synovites des mains, des poignets et des genoux, une fièvre à 40°, une altération marquée de l'état général avec marche impossible et fonte musculaire importante, et des adénopathies cervicales et inguinales dures mobiles de 1,5 cm de grand axe. Les examens biologiques montraient un syndrome inflammatoire majeur (protéine C réactive à 300 mg/l, vitesse de sédimentation à 80 mm la première heure) associé à une hyperleucocytose à 15000 éléments/mm^3^ dont 88% des polynucléaires neutrophiles, une anémie inflammatoire (hémoglobine à 8,5 g/dl). La ponction du liquide articulaire mettait en évidence un liquide inflammatoire, stérile et sans cristaux (9000 éléments/mm^3^ dont 80% de polynucléaires neutrophiles). La recherche d'une maladie infectieuse était négative: des séries d'hémocultures au moment des pics fébriles, l'examen cytobactériologique des urines, le bilan phtysiologique (recherche de bacille tuberculeux dans les expectorations et l'intradermo-réaction), les sérologies virales (HIV, hépatite B et C et syphilis), la ponction lombaire, la radiographie thoracique et l'échographie cardiaque à la recherche d'une endocardite. Malgré la négativité du bilan infectieux et devant la persistance de fièvre, un traitement antibiotique probabiliste était instauré mais sans efficacité.Une enquête plus approfondie était effectuée à la recherche d'une pathologie néoplasique notamment lymphomateuse: une tomodensitométrie cervico-thoraco-abdomino-pelvienne (TDM C-TAP) objectivait des adénopathies cervicales bilatérales infra-centimétriques, des multiples adénopathies axillaires et inguinales avec splénomégalie. La biopsie de l'adénopathie axillaire était en faveur d'une adénite réactive. Dans le cadre d'une maladie auto-immune: anticorps antinucléaires et facteur rhumatoïde étaient négatifs. Le diagnostic de la maladie de Still a été suspecté et on a complété par le dosage de la ferriténémie revenait élevée à 4787 dont la fraction glycosylée représentait 15%. Le tableau clinique s'enrichissait par l'apparition de rash cutané rose saumon maculo-papuleux et prurigineux (Figure 1) au moment des pics fébriles et qui disparaissait après 5jours. Le tableau de présentation remplissait les critères diagnostiques de la maladie de Still de l'adulte selon Yamaguchi et Fautrel (Figure 2), et un traitement par glucocorticoïde à base de prednisolone à forte dose (60 mg/j) était instauré pendant 4semaines puis dégression progressive. L'évolution était marquée par la disparition de la fièvre dès l'introduction de prednisolone, la régression des synovites dès la deuxième semaine de corticothérapie, la disparition des adénopathies inguinales à la troisième semaine, et la régression du syndrome inflammatoire biologique après une semaine du début de la corticothérapie.

**Tableau 1:** Les principaux critères diagnostiques de la maladie de Still de l’adulte

Yamaguchi et coll.¹	Cush et coll.²	Fautrel et coll.³
Sensibilité: 93,5-96,2%	Sensibilité: 84%	Sensibilité: 80,6%
Spécificité: 92,1%		Spécificité: 98,5%
Critères majeurs	Critères majeurs (2 points)	Critères majeurs
Fièvre ≥ 39°C, ≥ 1semaine	Fièvre quotidienne > 39°C	Pics fébriles ≥ 39°C
Arthralgies ≥ 2semaines	Eruption cutanée évanescente	Arthralgies
		Erythème transitoire
Eruption cutanée typique	Lc>12000/mm³et VS>40 mm/h	PMN ≥ 80%
Lc ≥ 10000/mm³, avec ≥ 80% de PMN	Absence d’ANA et de FR	Ferritine glycosylée ≤ 20%
	Ankylose carpienne	Maux de gorge
Critères mineurs	Critères mineurs (1 point)	Critères mineurs
Maux de gorge	Maux de gorge (prodromique)	Rash maculopapuleux
Adénopathies significatives et/ou splénomégalie	Atteinte du système réticulo-endothélial ou perturbation des tests hépatiques	
Perturbation des tests hépatiques	Sérosite	
	Ankylose cervicale ou tarsienne	Lc ≥ 10000/mm³
Absence d’ANA et de FR	Début des symptômes < 35ans	
	Arthrite	
Critères d’exclusion		
Infections (sepsis, mononucléose, etc.)		
Néoplasies (lymphome, etc.)		
Maladies systémiques (PR, LES, vasculites systémiques, etc.)		
Diagnostic	Diagnostic	Diagnostic
5 critères, dont au moins 2 majeurs et 0 critère d’exclusion	MSA probable: 10 points sur une observation de 12 semaines	4 critères majeurs, ou 3critères majeurs et 2 critères mineurs
	MSA certaine: 10 points sur une observation de 6 mois	

Lc = leucocytes; VS = vitesse de sédimentation ; PMN= polymorphonucléaires sanguines; ANA = anticorps antinucléaires; FR = facteur rhumatoïde; LES = lupus érythémateux systémique; PR = polyarthrite rhumatoïde.

¹ Yamaguchi M, Ohta A, Tsunematsu T et al. A preliminary criterion for classification of adult Still’s disease. J Rheumatol. 1992;19:424-30

² Cush JJ, Medsger TA, Christy WC et al. Adult-onset Still’s disease: clinical course and outcome. Arthritis Rheum, 1987, 30, 186-194

³ Fautrel B, Zing E, Golmard JL et al. Proposal for a new set of classification criteria for adult onset Still disease. Medicine 2002; 81: 194-200

## Discussion

La spondylarthrite ankylosante est un rhumatisme inflammatoire chronique au cours duquel la fièvre au long cours est rarement présente [1]. Cette observation permet de rappeler que lorsque la fièvre au long cours se présente, il faut penser à l'association avec d'autres affections systémiques. Dans notre cas, la maladie de Still de l'adulte se rajoute à un tableau connu de Spondyloarthrite, ce qui nous pousse à penser à un lien physiopathologique entre les deux pathologies.L'association de spondylarthrite ankylosante et MSA est rarement rapportée dans la littérature. AKKOC et al rapportait une série de 4 patients atteints de spondyloarthrite se présentaient avec un tableau cliniques de la MSA [3]. Tous les patients présentaient une fièvre prolongée, un rash cutané fugace, mais aussi des arthrites et des rachialgies inflammatoires. Dans une série ancienne de 202 cas de maladie de Still juvénile, une sacroiliite probable ou définie a été détectée chez 24% de ces patients [4]. Bien que la présence de sacroiliite chez 9% des patients porteurs de MSA a été mentionné dans une grande revue synoptique d'articles disponible sur le web [5], mais il n'existe pas à l'heure actuelle des données formelles sur la prévalence da la sacroiliite dans la MSA. La physiopathogénie de la MSA et de la spondyloarthrite est en grande partie inconnue. Il y'a une forte prédisposition génétique associée à l'HLA B27 pour développer les différents types de spondyloarthrites. L'association de la MSA aux certains sous types de HLA était rapportée notamment HLA B17, B18, B35, et DR2 [6], mais aucune relation avec HLA B27 n'a été démontrée [3]. L'hypothèse d'agent infectieux comme facteur déclenchant a été soulevé dans les deux pathologies MSA et spondyloarthrite, surtout chez les patients à prédisposition génétique. Dans la spondylarthrite ankylosante le microbiote intestinal est suspecté d'être responsable de l'apparition de la maladie [7]. Quant à la MSA l'hypothèse d'une infection virale ou bactérienne agissant comme facteur déclenchant a souvent été évoquée, mais les preuves sont faibles. Dans quelques observations, le début de la MSA survient après une infection virale, bactérienne, voire parasitaire plus ou moins bien documentée [8].

## Conclusion

L'association de spondylarthrite ankylosante à la MSA est une association rare mais possible, elle doit être évoquée à chaque fois qu'un patient connu porteur de spondyloarthrite se présentant avec une fièvre au long cours avec polyadenopathie et syndrome inflammatoire biologique important en dehors de toute infection ou pathologie néoplasique. L'hypothèse d'un lien physiopathologique entre les deux pathologies reste encore à prouver.

## Conflits d’intérêts

Les auteurs ne déclarent aucun conflit d'intérêts.
